# Depth-dependent fluence compensation without *a priori* knowledge of tissue composition for quantitative ultrasound-guided photoacoustic imaging

**DOI:** 10.1117/1.JBO.30.7.076005

**Published:** 2025-07-12

**Authors:** David Qin, Xinyue Huang, Timothy Sowers, Donald VanderLaan, Stanislav Emelianov

**Affiliations:** a Georgia Institute of Technology and Emory University School of Medicine, Wallace H. Coulter Department of Biomedical Engineering, Atlanta, Georgia, United States; b Parker H. Petit Institute for Bioengineering and Bioscience, Atlanta, Georgia, United States; c Georgia Institute of Technology, George W. Woodruff School of Mechanical Engineering, Atlanta, Georgia, United States; d Georgia Institute of Technology, School of Electrical Engineering, Atlanta, Georgia, United States

**Keywords:** photoacoustics, ultrasound, fluence, compensation, quantitative, light attenuation

## Abstract

**Significance:**

Compensation for depth-dependent fluence without *a priori* knowledge of tissue composition is a crucial unmet need for quantitative photoacoustic imaging.

**Aim:**

We developed a method for estimating the effective optical attenuation coefficient of bulk tissue with composition and optical properties that are not known in advance, through combined ultrasound/photoacoustic imaging during mechanical displacement of tissue.

**Approach:**

Ultrasound/photoacoustic imaging was performed on a target embedded in biological media while applying tissue displacement to change the optical path. After compensation for geometry-dependent scattering of light from light source apertures, the change of photoacoustic amplitude against optical path length was used to estimate the effective optical attenuation coefficient.

**Results:**

Using the developed approach, the estimation of the effective optical attenuation coefficient of tissue-mimicking (milk/water) phantoms and *ex vivo* porcine muscle and chicken breast was accurate compared with ground-truth literature values.

**Conclusions:**

Regardless of the varying geometries used for light delivery in photoacoustic imaging, it is feasible to perform ultrasound-guided photoacoustic imaging with simultaneous mechanical displacement of tissue to determine the effective optical attenuation coefficient of bulk tissue along the light path to the target.

## 1 Introduction

Photoacoustic (PA) imaging has potential for various preclinical and clinical applications such as cancer diagnosis, treatment monitoring, and assessment of vascularity and hemodynamics.^1^^,^^2^ This noninvasive modality uses nanosecond pulsed laser illumination of tissue, followed by absorption of light energy, thermoelastic expansion, and the emission of a broadband acoustic wave. This acoustic signal can then be detected by conventional ultrasound (US) hardware to produce an image with molecular contrast. Quantitative PA imaging uses multiwavelength PA imaging together with the known absorption spectra of various chromophores to estimate their concentration in tissue.^3^ These chromophores can be either endogenous such as hemoglobin,^4^ melanin,^5^ collagen, or exogenous such as gold nanoparticles or dyes,^6^^,^^7^ with applications such as early detection of lymph node metastasis, longitudinal monitoring of hypoxia in solid tumors, or tracking of nanoparticle-labeled cells in the tissue.^8^^–^^11^ These applications require accurate quantitative PA imaging to avoid incorrect classification of disease states or inaccurate counts of labeled cells *in vivo*. However, quantitative PA imaging relies on knowledge of tissue optical properties as well as the local fluence at every point in the image for accurate estimation of the local PA signal. Unfortunately, direct measurements of local fluence in the tissue require invasive probes,^12^ and estimation of local fluence remains an ill-posed inverse problem with many proposed solutions.^13^^,^^14^

Fluence compensation through estimation of local fluence or optical properties has been proposed in many different forms. The radiative transport equation (RTE) describes light transport in biological media, though analytical solutions to the RTE through direct inversion are generally only available for homogeneous, simple media or in cases of very shallow imaging.^15^^,^^16^ However, most cases of preclinical and clinical PA imaging involve greater depths and/or heterogeneous media, for which local fluence is an ill-posed inverse problem with no unique solution. Several computational approaches have been proposed to estimate fluence. Monte Carlo (MC) models are considered the gold standard in estimating light distribution in biological tissues.^17^^–^^22^ However, Monte Carlo methods are computationally expensive, and therefore, alternative approaches using iterative solvers for the RTE have seen interest.^23^^–^^25^ There has also been a focus on deep learning methods, given the difficulty in achieving direct analytical solutions.^26^^–^^30^ Taken together, these computational methods have formed the basis of attempts to compensate experimental data with modeled estimates of fluence.

Other attempts at fluence compensation have involved analytical methods, including iterative and noniterative optical inversion schemes to recover the absorption coefficient.^15^^,^^31^^–^^33^ These analytical solutions largely derive from diffusion theory and are relevant for narrow (pencil beam) geometries in highly scattering media,^34^ and from Beer–Lambert law for use with highly absorbing media.^35^ Experimentally, the recovery of $\mu_{s}$, $\mu_{a}$, and $\mu_{\mathrm{eff}}$ using diffusion theory and Beer–Lambert from a narrow-beam setup have been demonstrated.^36^ Others have combined experimental measurements of surface fluence with a finite-element model of fluence distribution in the tissue based on the diffusion approximation to the RTE to perform local fluence compensation or used Beer–Lambert with image analysis of artifacts to correct for depth-dependent attenuation.^37^^,^^38^ Finally, Kim et al.^39^ proposed an experimental method to use different subapertures to deliver narrow-beam light at different distances to a target, as a multiple irradiation method to determine fluence attenuation in a turbid medium. However, there is a need to expand the analytical framework to larger light delivery geometries that are presently used in preclinical and clinical PA imaging.

Many of the above methods rely on *a priori* knowledge of tissue optical properties. This is a problematic assumption as optical properties depend on measurement method, tissue type/hydration, and blood content/oxygenation. These properties may vary from one day to the next, even for the same subject.^40^^,^^41^ In addition, tissue properties are dependent on the methods used for tissue preparation and measurement.^42^^,^^43^ The high variability of optical properties in the literature has been shown, varying over time, sampling location, external temperature, gender, age, and skin pigmentation of the subject; published figures based on *ex vivo* tissues have even larger variations.^44^ These difficulties are compounded by the reality of *in vivo* PA imaging in complex, heterogeneous tissue structures with optical properties that do not line up with published values for homogeneous tissues measured *ex vivo* in isolation. Other challenges include the long computation time of MC and deep-learning models, obviating their use in real-time PA imaging scenarios.^30^ Together, this highlights the need for real-time fluence compensation methods that can be integrated into the typical PA imaging workflow, without prior knowledge of optical properties for the tissue under imaging for a particular subject and time.

Accordingly, we present an approach to estimating the optical attenuation coefficient that does not require *a priori* knowledge of tissue composition. A continuous ultrasound/photoacoustic (US/PA) acquisition is performed during simultaneous mechanical tissue displacement, causing a change in optical path length, which induces a change in the observed PA intensity at the target. This relationship can be used to deduce the optical attenuation coefficient of the bulk tissue along the light path without knowledge of its composition, but only after compensation for geometric spread (“sidescatter”) of light that is dependent on light source geometry.^12^ To address this, we perform one-time MC modeling to derive a generalizable compensation factor for the geometry-dependent scatter of light for a variety of apertures and tissues, showing that the compensation factor does not depend on exact tissue identity. Finally, we combine our measurements of target PA amplitude data with a geometry compensation factor from MC modeling. The resulting geometry-compensated PA data allow the facile estimation, in accordance with diffusion theory, of the effective optical attenuation coefficient of bulk tissue along the light path to the target. This method can be performed in real time in advance of a PA acquisition, and the recovered effective optical attenuation coefficient is used to normalize PA signals across different depths in that acquisition for more accurate quantitative PA imaging.

## 2 Materials and Methods

### 2.1 Theory

The photoacoustic signal at a location r is given by $P(r)=\Gamma \mu_{a}(r)\phi (r)$, where Γ is the Grüneisen parameter, $\mu_{a}$ is the absorption coefficient, and ϕ is the local fluence. The Grüneisen parameter Γ is assumed to be spatially invariant, whereas spatial variation of $\mu_{a}(r)$ is taken to be small. These assumptions lead to a linear dependence of observed PA amplitude P(r) on the fluence ϕ(r).

The analytical expression for ϕ(r) in a pencil beam geometry, derived from diffusion theory, can then be used as the starting point. It is well known that the propagation of light energy is exponential with depth, as given by the Beer–Lambert law for absorbing media, $\phi (r)=\phi_{0}\;\exp(-\mu_{a}r)$, where $\mu_{a}$ is the absorption coefficient and r is the radial distance from the source. Biological tissues are highly scattering, thereby requiring the incorporation of diffusion theory; for an isotropic point source within a semi-infinite homogeneous scattering medium, the 3D Green’s function for the fluence is then

$$\phi (r)=\frac{1}{4\pi Dr}\phi_{0}\;\exp(-\mu_{\mathrm{eff}}r)\propto (\frac{c}{r})\phi_{0}\;(-\mu_{\mathrm{eff}}r),$$ (1)

where D is the diffusion constant, c is a constant, the effective attenuation coefficient $\mu_{\mathrm{eff}}=\sqrt{3\mu_{a}(\mu_{a}+(1-g)\mu_{s})}$, $\mu_{s}$ is the scattering coefficient, and g is the scattering anisotropy.^45^^,^^46^ The corresponding solution for a semi-infinite collimated plane beam incident on the surface of the scattering medium at depth r=0 is the 1D Green’s function

$$\phi (r)=\frac{\mu_{\mathrm{eff}}}{2\mu_{a}}\phi_{0}\;\exp(-\mu_{\mathrm{eff}}r)\propto \phi_{0}\;\exp(-\mu_{\mathrm{eff}}r).$$ (2)

Although both forms of the Green’s function have an exponential decay component parametrized by $\mu_{\mathrm{eff}}$, they differ in the preceding term. The 3D case in Eq. (1) features a preceding geometric diffusion component $\left(\frac{c}{r}\right)$, which depends on the optical properties of the medium but also takes different forms based on the source geometry. The 1D case in Eq. (2) has no geometry term in front as there is no geometric loss with distance from a plane source. Equations (1) and (2) can therefore be generalized to

$$\phi (r)\propto (\frac{c}{r}{)}^{b}\phi_{0}\;\exp(-\mu_{\mathrm{eff}}r),$$ (3)

where b=1 for high scattering and b=2 for high absorbing media for an isotropic point source, and b=0 for a plane source in both scattering and absorbing conditions.^47^ The expression for fluence in Eq. (3) has two unknowns: $\mu_{\mathrm{eff}}$ and a geometry parameter b, requiring an estimate of b for a given geometry so that $\mu_{\mathrm{eff}}$ can be recovered.

Although b is given for analytical solutions to ideal point and plane sources, it is not known for beam geometries of finite extent, such as a rectangular fiber optic output, wherein light energy experiences sidescatter past the geometric extent of the output aperture.^12^ This necessitates an extension from analytical methods. Equation (3) can be rearranged to solve for b as a power law function for a light source with an arbitrary geometry

$${\left(\frac{c}{r}\right)}^{b}\propto \frac{\phi (r)}{\phi_{0}\;\exp(-\mu_{\mathrm{eff}}r)}.$$ (4)

We utilize Monte Carlo (MC) modeling to estimate b for a light source of an arbitrary geometry, using knowledge of the $\mu_{\mathrm{eff}}$ input into the model and the fluence distribution ϕ from the model output. This value of b is intrinsic to that geometry and can be used to recover

$$\mu_{\mathrm{eff}}\propto -r\;\ln(\frac{\phi (r)(\frac{1}{r}{)}^{-b}}{\phi_{0}}).$$ (5)

For estimation of $\mu_{\mathrm{eff}}$ in tissue, we propose to perform US/PA imaging during simultaneous displacement of tissue to change the optical path length to an embedded target. This method will allow the measurement of target PA amplitude over a change in the optical path length from the fiber output to the target. Under the assumption of tissue incompressibility and minimal change to optical properties during a small displacement, the PA amplitude at distance r from the transducer is given by $P(r)=(\frac{c}{r}{)}^{b}P_{0}e^{-\mu_{\mathrm{eff}}r}$. Imaging before and after a small displacement of the tissue, and comparing the ratio of the two measurement results in

$$\frac{P(r_{1})}{P(r_{2})}=\frac{(\frac{c}{r_{1}}{)}^{b}P_{0}e^{-\mu_{\mathrm{eff}}r_{1}}}{(\frac{c}{r_{2}}{)}^{b}P_{0}e^{-\mu_{\mathrm{eff}}r_{2}}}={\left(\frac{r_{2}}{r_{1}}\right)}^{b}e^{-\mu_{\mathrm{eff}}(r_{1}-r_{2})}.$$ (6)

The PA amplitudes $P(r_{1})$, $P(r_{2})$ and target locations $r_{1}$, $r_{2}$ are known from US/PA imaging, whereas b is taken from MC modeling of a fiber output with matching dimensions. Equation (6) can then be solved for $\mu_{\mathrm{eff}}$ from the experimental data, giving the optical attenuation of the bulk tissue along the light path.

### 2.2 Simulations

Monte Carlo modeling simulates the propagation of large numbers of photons from an arbitrary source through a volume for which optical properties ($\mu_{a},\mu_{s},g,n)$ are defined. We validated the utility of MC modeling to estimate the geometry parameter b for scattering-dominant media such as biological tissues, starting with isotropic point source and semi-infinite plane wave geometries for which b is known analytically from Eqs. (1) and (2) (Fig. S1 in the Supplementary Material). To model the light distribution of an isotropic point source, we specified an isotropic point source in the center of the volume and obtained the fluence throughout the entire modeled volume. Fluence ϕ[r] was taken along a vector in the z-direction extending out from the source to the edge of the volume. For the semi-infinite plane wave geometry, we modeled a large planar light source with the dimensions of one face of the cubic volume, placing this light source outside the volume. We then obtained ϕ[r] along a vector in the z-direction extending normally from the center of this plane into the volume.

**Fig. 1:**
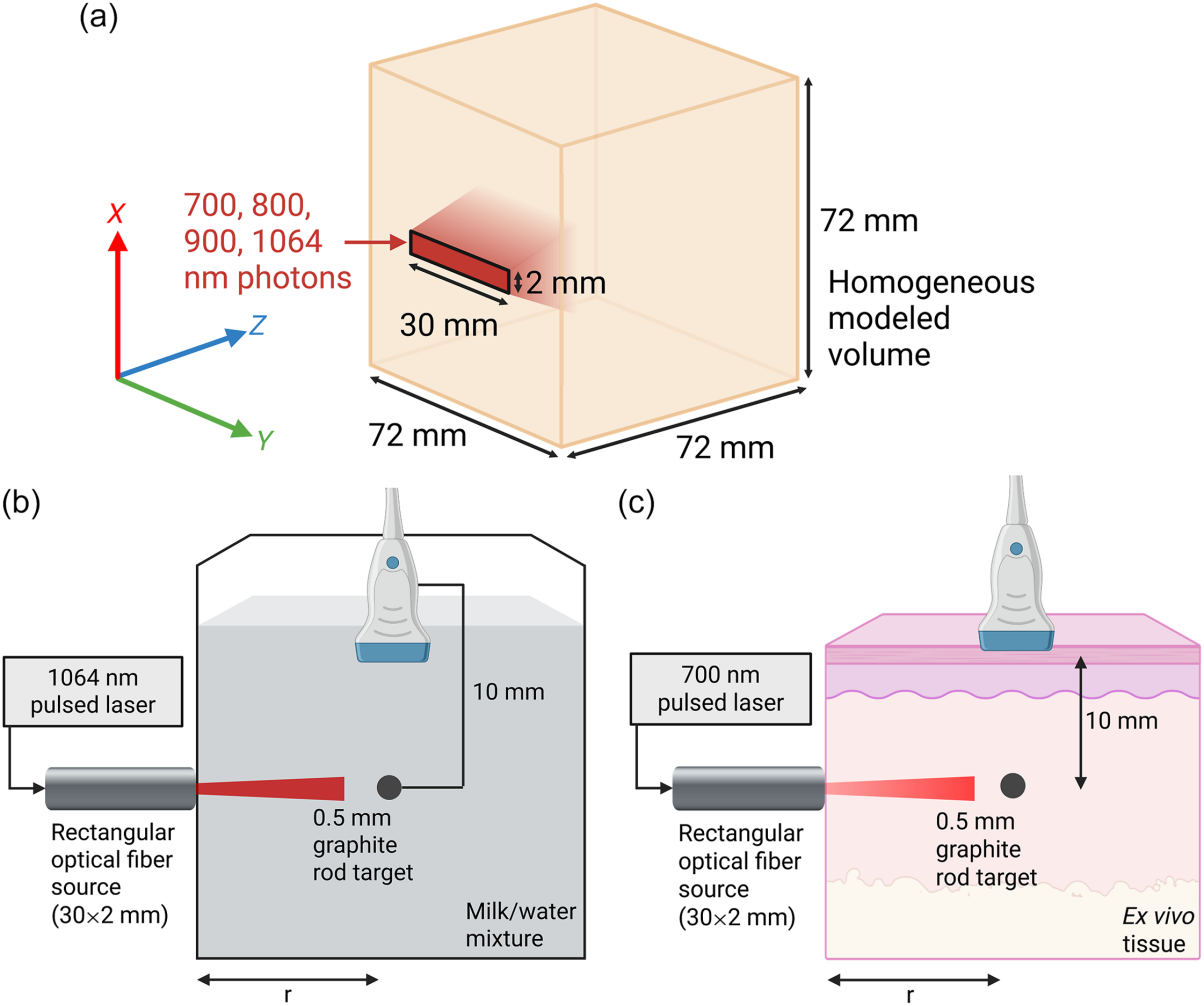
Setup of Monte Carlo modeling, liquid phantom and *ex vivo* experiments: (a) MC models used a 30×2  mm rectangular planar light source on one face of a 72×72×72  mm homogeneous volume with varying optical properties and input light wavelengths, with fluence taken along the z-direction extending out from the centerline of the rectangular light source in the x-direction. (b) Liquid phantom experiment utilized varying milk/water mixtures, a graphite rod target held at a constant position in the field-of-view of the MS250 transducer, and the target/transducer assembly moved to varying distance r from the light source. (c)  *Ex vivo* experiment utilized similar setup, with embedded graphite rod target in porcine muscle or chicken breast tissue, while the tissue was mechanically displaced by the motion of the optical fiber from the left, thereby changing distance r to the target.

We subsequently performed MC modeling for more complicated geometries such as a rectangular fiber optic output that may be used for photoacoustic imaging. Modeling a representative rectangular planar light output (30  mm×2  mm) placed at one face of a homogeneous volume and radiating collimated light [Fig. 1(a)], we found fluence ϕ[r] in the z-direction from the centerline of the rectangular planar source. We also performed MC modeling for the same light source geometry in a multilayer tissue model of skin, fat, and muscle.

For both the ideal geometries and the rectangular planar source, we used the relation $(\frac{1}{r}{)}^{b}=\frac{\phi [r]}{\phi_{0}\;\exp(-\mu_{\mathrm{eff}}r)}$ to solve for b for each geometry. The value of $\mu_{\mathrm{eff}}$ is known from its component values of $\mu_{a}$, $\mu_{s}$, and g that defined the optical properties of the volume used for MC, whereas $\phi_{0}$ and ϕ[r] are known from the MC model output. We performed a moving window calculation of b with a window size of 20 voxels (equivalent to 2 mm). A power law fit over the window allowed us to attain the local value of b and assess whether it was consistent at different distances from the light source. We then used the estimated b to back-solve for $\mu_{\mathrm{eff}}$, confirming the validity of our approach to estimating the attenuation coefficient.

Monte Carlo simulations were performed using the MC Extreme package.^48^ We used MCXLAB v2021.2 running on MATLAB r2022a (Mathworks, Natick, Massachusetts, United States), utilizing a workstation PC with a Nvidia GTX1080 GPU. All modeled volumes were 72×72×72  mm with a cubic voxel size of 0.1 mm [Fig. 1(a)]. We modeled the volumes to have defined $\mu_{s}:\mu_{a}$ ratios with constant $\mu_{\mathrm{eff}}$ or to have optical properties of various tissues derived from the literature^49^ (Table 1). The light sources were isotropic point sources or rectangular planar light outputs (30  mm×2  mm), placed at the center of the volume. For the 1D plane source, we placed a 72  mm×72  mm planar light source along one face of the volume. To ensure the broad applicability of our estimation method, we performed modeling at four wavelengths commonly used in PA imaging (700, 800, 900, 1064 nm). In our simulations, $10^{8}$ photons were used and the fluence-rate taken as ϕ[r] used in our calculations. Tissue volumes were homogeneous, except for the multilayer model (Fig. S5 in the Supplementary Material), which used 1 mm skin, then 2 mm fat, followed by muscle tissue.

**Table 1:** Optical properties used in MC modeling.

Tissue type	Wavelength (nm)	Absorption coefficient (${\mathrm{mm}}^{-1}$)	Scattering coefficient (${\mathrm{mm}}^{-1}$)	Anisotropy coefficient	Index of refraction
Skin	700, 800, 900, 1064	0.048, 0.043, 0.033, 0.020	14.3, 15.9, 16.8, 16.8	0.9	1.4
Fat	700, 800, 900, 1064	0.127, 0.108, 0.095, 0.079	23.0, 20.2, 18.5, 16.9	0.9	1.4
Muscle	700, 800, 900, 1064	0.048, 0.028, 0.032, 0.051	8.18, 7.04, 6.21, 5.73	0.9	1.4
0.001 $\mu_{s}:\mu_{a}$	700	0.289	0.00029	0.9	1.4
100 $\mu_{s}:\mu_{a}$	700	0.087	8.71	0.9	1.4
500 $\mu_{s}:\mu_{a}$	700	0.041	20.2	0.9	1.4

### 2.3 Experimental Validation

To validate our approach for the estimation of $\mu_{\mathrm{eff}}$ of biological media, we performed combined ultrasound/photoacoustic (US/PA) imaging of a graphite rod target while it was displaced within a scattering medium of dilute milk in water [Fig. 1(b)]. First, we affixed a graphite rod (0.5 mm diameter) at a stationary location within the field of view of a 256-element, 23 MHz center frequency linear US transducer (Vevo MS250, Visualsonics, Toronto, Canada) connected to a Vevo 2100 ultrasound system. The US transducer and graphite rod target were immersed in a tank filled with 500 mL of degassed water and varying concentrations of fat-free milk (2% through 25% v/v). A fiber optic (30  mm×2  mm aperture size) was placed on the outside of the clear plastic tank, positioned perpendicularly to the axis of imaging. We used a Tempest-10 Nd:YAG laser (New Wave Research Inc., Fremont, California, United States) to provide 1064 nm pulsed laser light (pulse width 3 to 5 ns, pulse repetition frequency 10 Hz). US/PA imaging was performed on the Vevo 2100 system, whereas a linear translation stage (UTS 150 PP, Newport Corp., Irvine, California, United States) was used to slowly move (0.25  mm/s) the combined graphite rod/US transducer assembly within the tank of liquid from 5 mm out to 30 mm distance from the fiber output. Conventional PA imaging on the Vevo 2100 was performed at 10 Hz. The data were downloaded and processed on a desktop workstation running MATLAB r2023a. The maximum PA amplitude P[r] at the target for each frame was determined and then plotted as a function of the distance r.

We assumed P[r] to take the form of $\left(\frac{1}{r}{)}^{b}\;\exp(-\mu_{eff}r\right)$. This relation was multiplied by $r^{b}$ to remove the geometric diffusion term, with r known from the position of the linear translation stage with respect to fiber output and b known from our previous MC modeling (Sec. 2.2). We then linearized the remaining exponential term $\exp(-\mu_{\mathrm{eff}}r)$ by taking the natural logarithm and performed a moving window linear fit (window size of 20 corresponding to 2 mm) to determine the local value of $\mu_{\mathrm{eff}}$. We performed this calculation over the entire range of depths to confirm the estimate of $\mu_{\mathrm{eff}}$ was consistent over depth. Ground-truth $\mu_{\mathrm{eff}}$ values of water and various water-milk combinations were calculated from the literature.^50^

We further confirmed our approach with *ex vivo* experiments. Using the same setup whereby imaging and light delivery axes were perpendicular [Fig. 1(c)], we performed imaging of a graphite rod target inserted into a piece of *ex vivo* porcine muscle. We used the same MS250 transducer and Vevo 2100 system, whereas the same fiber optic as before was attached to the Newport linear translation stage to move the fiber optic tip horizontally to displace the tissue. The fiber optic tip was placed in the US/PA imaging field of view so that its location could be tracked. While performing US/PA imaging with 700 nm, 10 Hz PRF laser output from an Nd:YAG laser with optical parametric oscillator (Vevo LAZR), we slowly moved (0.10  mm/s) the tip of the fiber optic horizontally into the porcine muscle, causing a tissue displacement of up to 20 mm and a change in the lateral optical path length to the graphite rod target. Using co-registered US/PA images, the location of the fiber tip was measured with respect to the location of the target to obtain the optical path length. The maximum PA amplitude at the target was measured, and a 30-frame smoothing window was applied to remove noise. Subsequently, the same calculation as above was performed to estimate $\mu_{\mathrm{eff}}$, using a 20-frame moving window (around 0.2 mm displacement). Finally, we confirmed our ability to estimate $\mu_{\mathrm{eff}}$ for chicken breast tissue as a different model, performing the same experiment at 700 nm for three different samples.

## 3 Results

### 3.1 Modeling

We first confirmed that Monte Carlo could be used to calculate the geometry parameter b, verified against analytical values for simple ideal geometries as given in Eqs. (1) and (2) [Fig. S1(A) in the Supplementary Material]. Modeling an isotropic point source radiating 700, 800, 900, and 1064 nm light, we found for all wavelengths that b=2 in an absorption-dominant case in which the $\mu_{s}:\mu_{a}$ ratio was 1:1000. Likewise, for the scattering-dominant case, b=1 when $\mu_{s}:\mu_{a}$ ratio was above 100:1. We found b=0 for the case of a semi-infinite plane source radiating into either absorption or scattering-dominated media [Fig. S1(D) in the Supplementary Material]. Finally, we verified that our calculated values of b could then be used to accurately recover $\mu_{\mathrm{eff}}$ of the medium [Figs. S1(C) and S1(E) in the Supplementary Material].

**Fig. 2:**
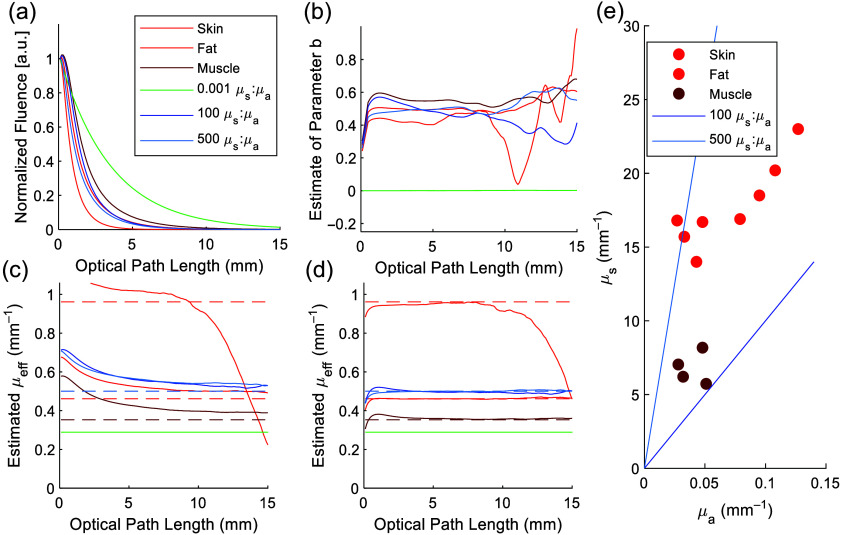
Monte Carlo simulation results of a rectangular 30  mm×2  mm aperture in a homogeneous volume with optical properties of skin, fat, muscle (red-shaded series) and of defined $\mu_{s}:\mu_{a}$ ratios (absorption-dominant in green-shaded series, scattering-dominant in blue-shaded series): (a) Normalized fluence as a function of distance from light source, along the normal axis extending out from the center of the planar light source. (b) Estimated value of geometry parameter as a function of distance from light source, showing that biological tissues (red) and high $\mu_{s}:\mu_{a}$ ratios (blue) converge to similar estimates, whereas the absorption-dominant case (green) behaves similarly to a semi-infinite plane wave. (c) Estimation of $\mu_{\mathrm{eff}}$ without compensation for geometry shows overestimation (solid lines) compared with the ground-truth (dotted lines). The absorption-dominant case recovers correctly due to the lack of broadening of the light beam in the absence of scatter. (d) Estimation of $\mu_{\mathrm{eff}}$ with geometry compensation of b=0.5 applied, showing correct estimation for all cases beginning at 1 mm from the light source. (e) Skin, fat, and muscle tissues at all four optical wavelengths used have $\mu_{s}:\mu_{a}$ ratios above 100.

Next, we performed the same analysis on MC modeling of the 30  mm×2  mm rectangular aperture. Light fluence increased to a subsurface fluence peak around 1 mm below the surface of the volume and then dropped substantially with depth [Fig. 2(a)]. Past the subsurface peak, we found that the geometry parameter could be clustered into two groups—cases in which $\mu_{a}\gg \mu_{s}$ and cases in which $\mu_{a}\ll \mu_{s}$; these are respectively notated as the green-shaded and blue-shaded data series in Fig. 2(b). Notably, the tissue data (red-shaded data series) cluster closely with the scattering-dominant media. However, the calculation of b became noisy at distances greater than 10 mm from the source due to the discrete nature of MC modeling and finite numbers of photons modeled, resulting in photon counts approaching zero at distances far from the source.

For the planar rectangular geometry, we found that b converged to roughly 0.5 in cases of high scattering ($\mu_{s}:\mu_{a}$ ratios above 100:1), regardless of the identity of the tissue [Fig. 2(b)]. Therefore, provided that the condition of high scattering is satisfied, as in all soft tissues, we were able to use this one MC modeling result for all of our subsequent estimation of $\mu_{\mathrm{eff}}$ without regard to tissue type in either modeling or experiments.

As photoacoustic imaging is commonly done with multimode fibers with a divergence angle, we also investigated the effect of the source numerical aperture (NA), finding a slight impact on b with increasing NA but still very close to 0.5 [Fig. S4(B) in the Supplementary Material], particularly for lower NA around 0.2 as seen on our fibers. Next, we recovered $\mu_{\mathrm{eff}}$ from the MC data either assuming b=0 or b=0.5. The former case represents overestimation of $\mu_{\mathrm{eff}}$ without compensation for geometry [Fig. 2(c)]. In the latter case, we stably recovered $\mu_{\mathrm{eff}}$ across depths from 1 to 15 mm for multiple homogeneous tissue types [Fig. 2(d)] as well as for the multilayer tissue model (Fig. S5 in the Supplementary Material). The estimate for fat $\mu_{\mathrm{eff}}$ was an exception past 10 mm as the high attenuation led to low photon counts deeper within the volume. Having validated our model-based approach to finding the geometry parameter for an arbitrary source, we also noted that all the modeled tissues had $\mu_{s}:\mu_{a}$ ratios above 100:1 at all wavelengths [Fig. 2(e)], such that they would fall into the highly-scattering group for which b=0.5. Finally, we also determined that the geometry parameter b was consistent laterally across the middle 15 mm of the field-of-view, with estimates of $\mu_{\mathrm{eff}}$ also accurate in the middle section of the imaging plane (Fig. S3 in the Supplementary Material), suggesting the method is robust for targets situated in the center and up to 7.5 mm off-axis, with reductions in the accuracy of estimation toward the edges of the rectangular light source in the lateral direction.

### 3.2 Experimental Validation

**Fig. 3:**
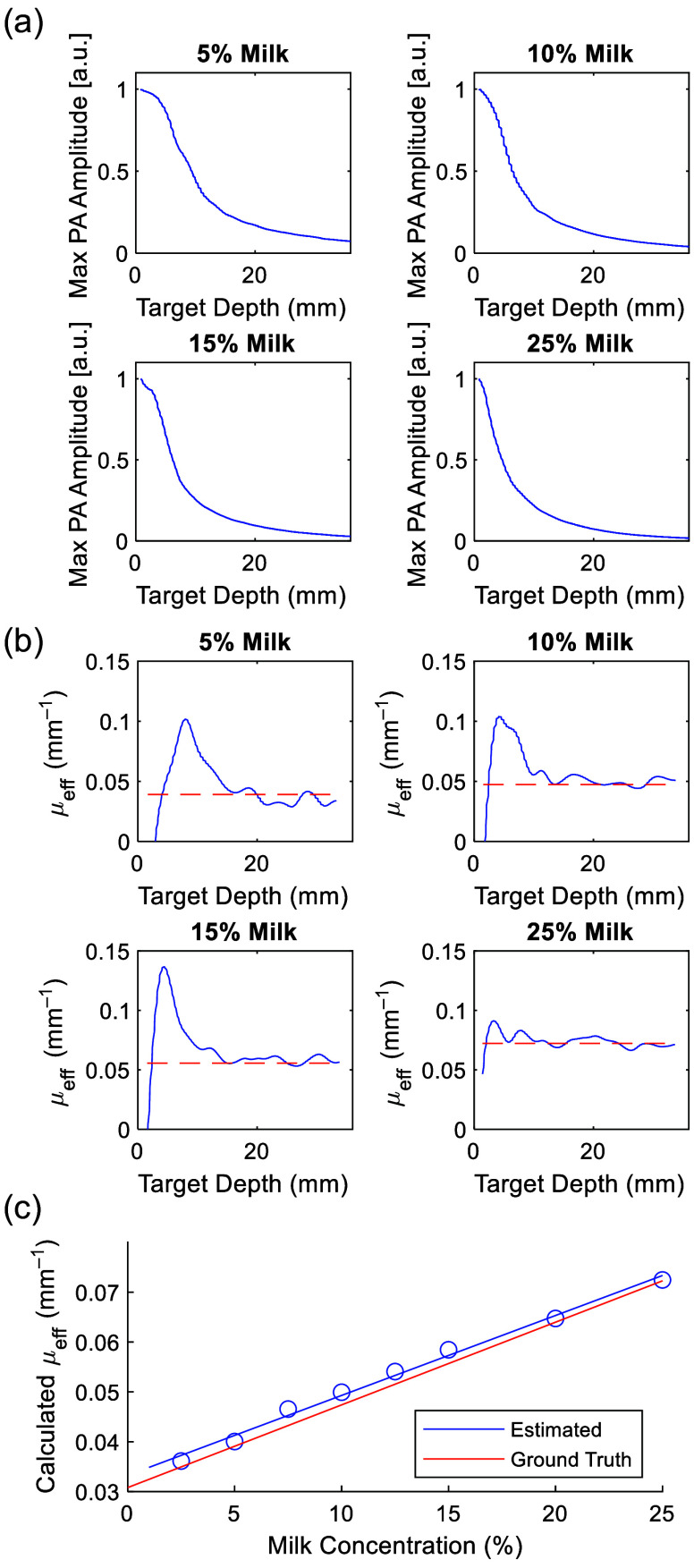
Estimation of $\mu_{\mathrm{eff}}$ of a milk/water mixture: (a) Four representative plots of normalized maximum PA amplitude at the target as a function of distance from the fiber output within the milk bath. (b) Estimation of $\mu_{\mathrm{eff}}$ (blue solid line) agrees with ground-truth (orange dashed line) past 15 mm. (c) Estimated $\mu_{\mathrm{eff}}$ plotted as a function of milk concentration shows linear correlation ($R^{2}=0.9825$) and close agreement with the ground-truth. Estimation error is within 8% of the true value.

Our experiments confirmed the validity of our approach. We imaged a graphite rod at varying depths, obtaining maximum PA amplitude values for different milk concentrations ranging from 2.5% to 25% v/v; four representative plots are shown in Fig. 3(a). Our approach showed an overestimate in the near-field close to the light source before converging to a steady value at greater depths [Fig. 3(b)]. The depth at which this convergence occurred varied; for lower milk concentrations, the estimate converged by 15 mm, whereas convergence occurred at shallower depths down to 5 mm for higher milk concentrations. The lower concentration samples had lower scattering, likely requiring longer distances for photons to become diffuse. In the near field, their ballistic behavior likely resulted in an overestimate of $\mu_{\mathrm{eff}}$. By contrast, higher concentrations of milk caused photons to become fully diffuse over a shorter optical path, making $\mu_{\mathrm{eff}}$ estimation accurate at a shallower range. In the constant regime past the shallow peak (15 to 30 mm), we estimated an average $\mu_{\mathrm{eff}}$ value that agreed within 8% of ground-truth values reported in the literature for the same combination of water and milk used in our phantom [Fig. 3(c)].^50^ In addition, plotting the estimates of $\mu_{\mathrm{eff}}$ as a function of milk concentration showed a strong linear correlation ($R^{2}=0.98$). Changing the window size used for $\mu_{\mathrm{eff}}$ estimation showed a mixed trend with respect to estimation error and decreasing variance (Fig. S2 in the Supplementary Material), suggesting that larger window sizes result in more steady estimates. For the 25% milk concentration, error decreased rapidly by 2 mm window size, suggesting that for higher scattering media such as realistic biological tissues, 2 mm displacement was sufficient for accurate estimation.

**Fig. 4:**
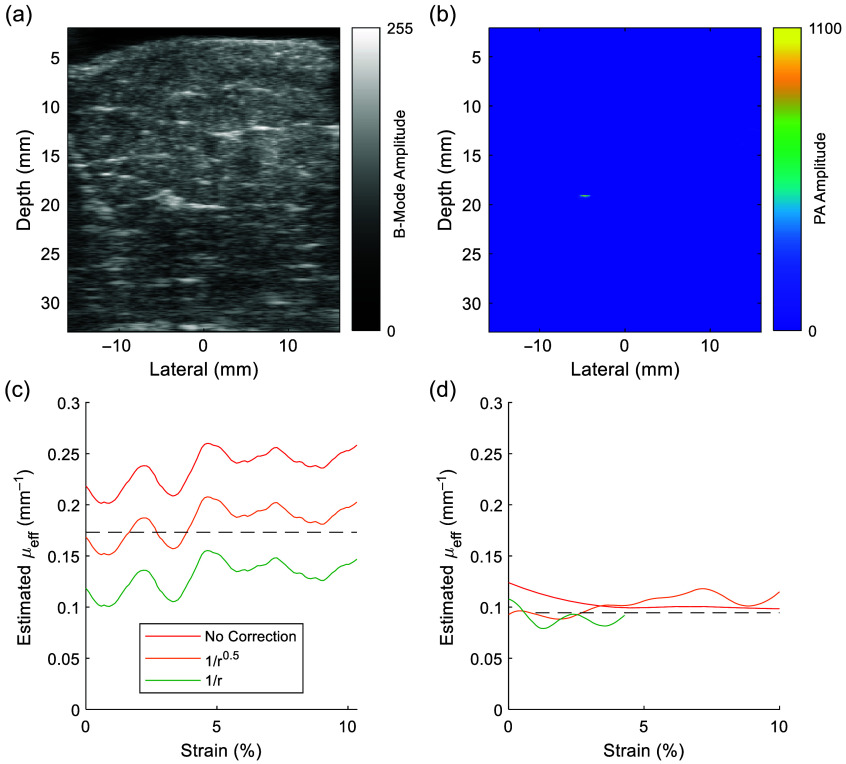
Estimation of $\mu_{\mathrm{eff}}$ of *ex vivo* porcine muscle and chicken breast: (a) US image of *ex vivo* porcine muscle. (b) PA image showing strong signal from embedded graphite rod. (c) In *ex vivo* porcine tissue, estimation of $\mu_{\mathrm{eff}}$ is possible over a small displacement up to 10% strain. Inaccurate estimate of b=0 will lead to an overestimate of $\mu_{\mathrm{eff}}$, whereas b=1 will lead to an overestimate of $\mu_{\mathrm{eff}}$. (d) For n=3 samples of *ex vivo* chicken breast tissue, the geometry-corrected estimation of $\mu_{\mathrm{eff}}$ is accurate and consistent over a small displacement up to 10% strain.

The *ex vivo* experiments provided evidence that our approach can also work in solid tissues. As we used the same optical fiber as in the previous experiment, we used the same matched value of b=0.5 from our modeling to estimate $\mu_{\mathrm{eff}}$. We performed combined US/PA imaging (Figs. 4(a) and 4(b)] and estimated $\mu_{\mathrm{eff}}$ as a function of strain ($\frac{\Delta \text{Length}}{\text{Length}}$), finding that the mean $\mu_{\mathrm{eff}}$ of a representative slab of porcine muscle tissue agreed with values reported in the literature^51^ within around 10% error [Fig. 4(c)]. Estimates of $\mu_{\mathrm{eff}}$ were sensitive to noise and small fluctuations in depth, so we applied frame-averaging, as described in Sec. 2.3. We also explored the effects of an incorrect estimate of b on the calculation of $\mu_{\mathrm{eff}}$, finding that the value of b shifted the estimate of $\mu_{\mathrm{eff}}$. Using b=0 (no compensation) resulted in an overestimate of $\mu_{\mathrm{eff}}$, which was expected due to geometric spread being erroneously attributed to optical attenuation, whereas b=1 resulted in an under-estimate due to the higher amount of assumed geometric spread [Fig. 4(c)]. Finally, we confirmed our ability to estimate $\mu_{\mathrm{eff}}$ in a different, less-attenuating tissue of chicken breast, finding accurate and steady estimation up to 10% strain [Fig. 4(d)].

## 4 Discussion

We have combined simulation with experiments to develop a method for the determination of the effective optical attenuation coefficient without prior knowledge of tissue composition. A key part of our approach is the calculation of a geometry parameter for an arbitrary aperture, which is an extension from exact, analytical values given for ideal isotropic and semi-infinite planar light sources. In our modeling, we found that the $\mu_{s}:\mu_{a}$ ratio determined the value that b converged to as the distance from the light source increased [Fig. 2(b)]. Ordinarily, this would require *a priori* knowledge of the optical properties of the tissue, invalidating our goal of obtaining tissue $\mu_{\mathrm{eff}}$. However, in a survey of literature values of various biological tissues,^42^^,^^49^ we found that their $\mu_{s}:\mu_{a}$ ratios all exceeded 100:1 [Fig. 2(e)], in agreement with the common rule-of-thumb that biological tissues are heavily scattering.^42^^,^^52^ Therefore, modeling of generic tissue with defined $\mu_{s}:\mu_{a}$ ratios (over 100:1) was adequate for generating a geometry parameter broadly generalizable to a variety of biological tissues, thereby lessening the requirement for prior knowledge of tissue optical properties or composition. In addition, the parameter value can be considered an intrinsic property of that light delivery aperture. Once calculated through one instance of MC modeling of a scattering-dominant volume, this value may be used for the estimation of $\mu_{\mathrm{eff}}$ in heterogeneous tissue without requiring knowledge of tissue identity.

We have used MC modeling for a generalized geometry parameter to encapsulate the optical problem. In practice, acoustic factors such as transducer bandwidth, acoustic attenuation, and measurement noise will affect the sensitivity of PA detection. However, we have found that acoustic variation over a few millimeters of axial or lateral displacement (as in our work) has a negligible impact on the PA amplitude, in contrast to the exponential change in PA amplitude due to optical attenuation.

Our method of mechanically displacing the tissue during simultaneous US/PA imaging also offers advantages for real-time fluence compensation. Measuring the PA amplitude change of a target during a change in optical path length induced by displacement is a simple method of estimating the attenuation experienced by light during propagation from the fiber output to the target. The estimated $\mu_{\mathrm{eff}}$ is specific to the bulk tissue in the field of view and is relevant even in the case of multilayered tissues (Fig. S5 in the Supplementary Material). A similar displacement step can be performed at the start of every PA imaging session to account for variability between subjects and over time. Key to this result is the assumption that tissue optical properties remain unchanged during displacement. This assumption is held to be valid due to tissue incompressibility as has been demonstrated in the literature,^53^^–^^59^ whereby small displacements are not expected to cause tissue blanching (i.e., no change to blood volume and therefore tissue absorption) and scattering from solid tissue is expected to stay constant. Our *ex vivo* experiments showed the feasibility of recovering $\mu_{\mathrm{eff}}$ with as little as 2% strain, which is small and unlikely to result in significant changes to local tissue optical properties. We also highlight that in our *ex vivo* work, we were able to consistently and accurately estimate $\mu_{\mathrm{eff}}$ up to as much as 10% strain [Fig. 4(d)], suggesting that the optical properties along the light path did not change as the degree of displacement increased. Had the displacement been sufficient to change the optical properties of the tissue through blanching, our method would have also detected this through a decrease in the attenuation coefficient at very high strain.

Practically, MC modeling to determine the geometry parameter for a given aperture geometry only needs to be performed once (on any highly-scattering volume) and can take ∼20  min, whereas the experimental estimation of $\mu_{\mathrm{eff}}$ can be done quickly in near real time, immediately ahead of a PA imaging session. The highlight of our method is its simplicity and ability to provide a real-time, subject-specific estimation of $\mu_{\mathrm{eff}}$ that is easily integrated into a conventional PA imaging workflow; it requires no training data, knowledge of the tissue being imaged, or chromophore calibration. This is in contrast to other recent works on fluence correction that have variously used well-characterized phantoms and digital equivalents to train U-Nets to find spatially correlated $\mu_{\mathrm{eff}}$ estimates for the entire field-of-view, as well as model-based inversion techniques that use arteries as reference fluence markers to generate a fluence field without needing a large training dataset.^28^^,^^60^ We believe our method is complementary to these other approaches and can be used to sample fluence maps experimentally as inputs or verification for the aforementioned techniques.

Notably, the proposed method can only be used to determine tissue $\mu_{\mathrm{eff}}$, which represents attenuation from all sources, and not its component values $\mu_{a}$, $\mu_{s}$, g, and n. As others have discussed, simultaneous and unique estimation of $\mu_{a}$, $\mu_{s}$ from an acquisition is an ill-posed problem.^24^^,^^61^ However, knowledge of $\mu_{\mathrm{eff}}$ for the tissue in the field of view is useful in equalizing relative PA amplitudes at different depths [Eq. (6)]. Therefore, this could be a simple approach to improving the accuracy of quantitative photoacoustic imaging at depth.

A limitation of our work was the perpendicular light delivery setup. We used a fiber optic with a rectangular aperture to deliver light perpendicular to the imaging axis as it was possible to calculate a geometry parameter for this rectangular aperture. However, US/PA imaging is typically done with US transducers integrated with fiber outputs that flank the imaging plane in the elevational direction, such as the LZ250 US/PA transducer used with the Vevo 2100 system. This is a complicated geometry with a complex light distribution in the elevational direction as others have shown, where the light pattern is distinct even in highly scattering conditions.^62^^,^^63^ Further work is needed to ascertain a method to determine a geometry parameter for such complicated cases where multiple light fields pass obliquely through the imaging plane, possibly through experimental measurement of the optical light field in media of varying absorption/scattering ratios to yield a three-dimensional look-up table. ^37^ Alternatively, our method could be used with newly developed transparent transducers capable of coplanar US/PA imaging, leveraging the fact that a simple rectangular aperture could be used to deliver light from behind the US transducer.^64^^,^^65^ Such alternatives could be used for real-time fluence compensation in *in vivo* imaging cases for which this perpendicular experimental setup is unrealistic as our method of compensating for geometric sidescatter from a single planar aperture is readily applicable to these transducers.

Our work also only utilized 1064 nm laser illumination for the phantom experiment and 700 nm laser light for the *ex vivo* experiments. However, the phenomenon of spectral coloring is well known for multiwavelength PA imaging, where wavelength-dependent attenuation of light causes a shift in the spectra of light at deeper voxels compared to the spectra at the surface.^66^ Spectral coloring is a well-studied challenge to accurate photoacoustic imaging, and attempts have been made to correct for it.^67^ A possible extension of our method could be used at multiple wavelengths to determine $\mu_{\mathrm{eff}}$ as a function of wavelength for a particular slice of tissue in view.

Finally, we utilized a graphite rod as a strong broadband absorber for our target, but conventional PA imaging will not generally have the benefit of a distinct exogenous target. Although preclinical applications could utilize exogenous contrast agents injected into the tissue at various depths to serve as local *in situ* targets, known anatomical features such as arteries could also be used as references in place of a graphite rod.^68^^,^^69^ Alternatively, the diffuse background PA signal in the image could be measured in conjunction with strain imaging to measure tissue displacements. This approach has the added benefit of being able to compensate for various complex displacements in tissue (including translation, rotation, and shear motion).^70^ A displacement map derived from strain imaging could be used to compensate a PA acquisition for motion, yielding accurate pixel-wise changes in signal for subsequent fluence correction. In this manner, a large region of interest could be used for the aggregate estimation of $\mu_{\mathrm{eff}}$.

Here, we have developed a method for the estimation of the effective optical attenuation coefficient of tissue during a PA imaging session without *a priori* knowledge of tissue composition. We utilized MC modeling to arrive at an approximation of the geometric diffusion of light emitted from an aperture of arbitrary size and shape, which was an extension of analytical solutions to ideal point and plane sources. This demonstrates the utility of Monte Carlo for the determination of an intrinsic geometry parameter for a source of any shape, generalizable to a wide range of tissues without prior knowledge of their composition. This could be useful for simple models of light distribution from fiber outputs of various geometries. In addition, we showed that displacement of tissue could be used to alter the optical path length from the light source to a target in the tissue, giving a relationship between PA amplitude and optical path length for estimating the optical attenuation coefficient. Furthermore, we highlight that this method is easy to perform and robust to changes in optical properties between subjects, parts of the body, and over time. Through real-time estimation of the effective optical coefficient for a slice of heterogeneous tissue, the amplitude of PA signals at depth can be adjusted relative to those closer to the surface, resulting in more accurate quantitative PA imaging. An example of this is in US/PA-mediated tracking of contrast agent-labeled cells within animal models, where knowledge of the attenuation coefficient could be used to improve the accuracy of estimates of cell count at various depths in the image. Finally, this work also illuminates the need for further work to determine the geometry parameters of even more complex light delivery geometries, such as those of integrated US/PA transducers in use. Finding these values would assist in the broader adoption of quantitative PA for preclinical and clinical imaging applications.

## 5 Conclusion

In this work, we developed a method for US/PA-mediated estimation of heterogeneous tissue optical properties without prior knowledge of tissue composition. We show that Monte Carlo modeling could be used to derive an intrinsic geometry compensation factor for an arbitrary aperture, broadly generalizable to a wide range of biological tissues, and used for the estimation of $\mu_{\mathrm{eff}}$. Our work utilized a scattering phantom of milk and water, as well as *ex vivo* porcine and chicken tissue to validate our method for determining $\mu_{\mathrm{eff}}$ via tissue displacement. Utilizing a perpendicular geometry for light delivery, we found that for small displacements, the estimation of $\mu_{\mathrm{eff}}$ was possible for milk/water combinations (over 2 mm) as well as *ex vivo* porcine muscle and chicken breast (over 0.2 mm). Combined with further work on elucidating the light distribution from more complicated geometries such as combined US/PA transducers, this work suggests that the effective attenuation coefficient of bulk tissue can be recovered *in situ* prior to an imaging session for immediate compensation of fluence at depth. Our work highlights the need for more investigation into experimental methods of finding tissue optical properties as an alternative to computational methods based on *a priori* assumptions of optical properties. Ultimately, this work could be extended in many directions with varied applications and light delivery geometries that are applicable to pre-clinical and clinical PA imaging.

## Supplementary Material

10.1117/1.JBO.30.7.076005.s01

## Data Availability

The code and data presented in this work are available upon reasonable request.

## References

[1] Wang L. V. Hu S., “Photoacoustic tomography: in vivo imaging from organelles to organs,” Science 335(6075), 1458–1462 (2012). SCIEAS 0036-8075 10.1126/science.1216210 22442475 PMC3322413

[2] Das D. et al., “Another decade of photoacoustic imaging,” Phys. Med. Biol. 66(5), 05TR01 (2021). PHMBA7 0031-9155 10.1088/1361-6560/abd669 33361580

[3] Cox B. T. et al., “Quantitative spectroscopic photoacoustic imaging: a review,” J. Biomed. Opt. 17(6), 061202 (2012). JBOPFO 1083-3668 10.1117/1.JBO.17.6.061202 22734732

[4] Laufer J. et al., “Quantitative spatially resolved measurement of tissue chromophore concentrations using photoacoustic spectroscopy: application to the measurement of blood oxygenation and haemoglobin concentration,” Phys. Med. Biol. 52(1), 141–168 (2006). PHMBA7 0031-9155 10.1088/0031-9155/52/1/010 17183133

[5] Mizukoshi K. Iwazaki H. Ida T., “Quantitative analysis of age-related changes in vascular structure, oxygen saturation, and epidermal melanin structure using photoacoustic methods,” Skin Res. Technol. 30(1), e13537 (2024). 10.1111/srt.13537 38174730 PMC10765365

[6] Cook J. R. Frey W. Emelianov S., “Quantitative photoacoustic imaging of nanoparticles in cells and tissues,” ACS Nano 7(2), 1272–1280 (2013). ANCAC3 1936-0851 10.1021/nn304739s 23312348 PMC3584228

[7] Weber J. Beard P. C. Bohndiek S. E., “Contrast agents for molecular photoacoustic imaging,” Nat. Methods 13(8), 639–650 (2016).1548-7091 10.1038/nmeth.3929 27467727

[8] Gerling M. et al., “Real-time assessment of tissue hypoxia in vivo with combined photoacoustics and high-frequency ultrasound,” Theranostics 4(6), 604 (2014). 10.7150/thno.7996 24723982 PMC3982131

[r9] Luke G. P. Emelianov S. Y., “Label-free detection of lymph node metastases with US-guided functional photoacoustic imaging,” Radiology 277(2), 435–442 (2015). RADLAX 0033-8419 10.1148/radiol.2015141909 25997030 PMC4627438

[r10] Nasri D. et al., “Photoacoustic imaging for investigating tumor hypoxia: a strategic assessment,” Theranostics 13(10), 3346 (2023). 10.7150/thno.84253 37351178 PMC10283067

[11] Jhunjhunwala A. et al., “In vivo photoacoustic monitoring of stem cell location and apoptosis with caspase-3-responsive nanosensors,” ACS Nano 17(18), 17931–17945 (2023). ANCAC3 1936-0851 10.1021/acsnano.3c04161 37703202 PMC10540261

[12] Kessel D., Photodynamic Therapy of Neoplastic Disease, CRC Press (1990). 1421614

[13] Cox B. T. Laufer J. G. Beard P. C., “The challenges for quantitative photoacoustic imaging,” Proc. SPIE 7177, 717713 (2009). PSISDG 0277-786X 10.1117/12.806788

[14] Tarvainen T. Cox B., “Quantitative photoacoustic tomography: modeling and inverse problems,” J. Biomed. Opt. 29(S1), S11509 (2023). JBOPFO 1083-3668 10.1117/1.JBO.29.S1.S11509 38125717 PMC10731766

[15] Ripoll J. Ntziachristos V., “Quantitative point source photoacoustic inversion formulas for scattering and absorbing media,” Phys. Rev. E 71(3), 031912 (2005). 10.1103/PhysRevE.71.031912 15903464

[16] Powell S. Cox B. T. Arridge S. R., “A pseudospectral method for solution of the radiative transport equation,” J. Comput. Phys. 384, 376–382 (2019). JCTPAH 0021-9991 10.1016/j.jcp.2019.01.024

[17] Wang L. Jacques S. L. Zheng L., “MCML—Monte Carlo modeling of light transport in multi-layered tissues,” Comput. Methods Programs Biomed. 47(2), 131–146 (1995). CMPBEK 0169-2607 10.1016/0169-2607(95)01640-F 7587160

[r18] Jacques S. L., “Coupling 3D Monte Carlo light transport in optically heterogeneous tissues to photoacoustic signal generation,” Photoacoustics 2(4), 137–142 (2014). 10.1016/j.pacs.2014.09.001 25426426 PMC4242914

[r19] Hochuli R. et al., “Quantitative photoacoustic tomography using forward and adjoint Monte Carlo models of radiance,” J. Biomed. Opt. 21(12), 126004 (2016). JBOPFO 1083-3668 10.1117/1.JBO.21.12.126004 27918801

[r20] Zheng S. et al., “Quantitative photoacoustic tomography with light fluence compensation based on radiance Monte Carlo model,” Phys. Med. Biol. 68(6), 065009 (2023). PHMBA7 0031-9155 10.1088/1361-6560/acbe90 36821863

[r21] Kirillin M. et al., “Fluence compensation in raster-scan optoacoustic angiography,” Photoacoustics 8, 59–67 (2017). 10.1016/j.pacs.2017.09.004 29034169 PMC5635250

[22] Pattyn A. et al., “Model-based optical and acoustical compensation for photoacoustic tomography of heterogeneous mediums,” Photoacoustics 23, 100275 (2021). 10.1016/j.pacs.2021.100275 34094852 PMC8167150

[23] Tarvainen T. et al., “Reconstructing absorption and scattering distributions in quantitative photoacoustic tomography,” Inverse Probl. 28(8), 084009 (2012). INPEEY 0266-5611 10.1088/0266-5611/28/8/084009

[r24] Brochu F. M. et al., “Towards quantitative evaluation of tissue absorption coefficients using light fluence correction in optoacoustic tomography,” IEEE Trans. Med. Imaging 36(1), 322–331 (2017). ITMID4 0278-0062 10.1109/TMI.2016.2607199 27623576

[25] Saratoon T. et al., “A gradient-based method for quantitative photoacoustic tomography using the radiative transfer equation,” Inverse Probl. 29(7), 075006 (2013). INPEEY 0266-5611 10.1088/0266-5611/29/7/075006

[26] Manwar R. et al., “Deep learning protocol for improved photoacoustic brain imaging,” J. Biophotonics 13(10), e202000212 (2020). 10.1002/jbio.202000212 33405275 PMC10906453

[r27] Madasamy A. et al., “Deep learning methods hold promise for light fluence compensation in three-dimensional optoacoustic imaging,” J. Biomed. Opt. 27(10), 106004 (2022). JBOPFO 1083-3668 10.1117/1.JBO.27.10.106004 36209354 PMC9547608

[r28] Gröhl J. et al., “Moving beyond simulation: data-driven quantitative photoacoustic imaging using tissue-mimicking phantoms,” IEEE Trans. Med. Imaging 43(3), 1214–1224 (2024). ITMID4 0278-0062 10.1109/TMI.2023.3331198 37938947

[r29] Bench C. Hauptmann A. Cox B. T., “Toward accurate quantitative photoacoustic imaging: learning vascular blood oxygen saturation in three dimensions,” J. Biomed. Opt. 25(8), 085003 (2020). JBOPFO 1083-3668 10.1117/1.JBO.25.8.085003 32840068 PMC7443711

[30] Liang Z. et al., “Deep learning acceleration of iterative model-based light fluence correction for photoacoustic tomography,” Photoacoustics 37, 100601 (2024). 10.1016/j.pacs.2024.100601 38516295 PMC10955667

[31] Jetzfellner T. et al., “Performance of iterative optoacoustic tomography with experimental data,” Appl. Phys. Lett. 95(1), 013703 (2009). APPLAB 0003-6951 10.1063/1.3167280

[r32] Zemp R. J., “Quantitative photoacoustic tomography with multiple optical sources,” Appl. Opt. 49(18), 3566–3572 (2010). APOPAI 0003-6935 10.1364/AO.49.003566 20563210

[33] Harrison T. Shao P. Zemp R. J., “A least-squares fixed-point iterative algorithm for multiple illumination photoacoustic tomography,” Biomed. Opt. Express 4(10), 2224–2230 (2013). BOEICL 2156-7085 10.1364/BOE.4.002224 24156078 PMC3799680

[34] Patterson M. S. Wilson B. C. Wyman D. R., “The propagation of optical radiation in tissue. II: optical properties of tissues and resulting fluence distributions,” Lasers Med. Sci. 6(4), 379–390 (1991). 10.1007/BF02042460

[35] Oshina I. Spigulis J., “Beer–Lambert law for optical tissue diagnostics: current state of the art and the main limitations,” J. Biomed. Opt. 26(10), 100901 (2021). JBOPFO 1083-3668 10.1117/1.JBO.26.10.100901 34713647 PMC8553265

[36] Moes C. J. M. et al., “Measurements and calculations of the energy fluence rate in a scattering and absorbing phantom at 633 nm,” Appl. Opt. 28(12), 2292–2296 (1989). APOPAI 0003-6935 10.1364/AO.28.002292 20555514

[37] Naser M. A. et al., “Improved photoacoustic-based oxygen saturation estimation with SNR-regularized local fluence correction,” IEEE Trans. Med. Imaging 38(2), 561–571 (2019). ITMID4 0278-0062 10.1109/TMI.2018.2867602 30207951 PMC6445252

[38] Park S. et al., “Compensation for non-uniform illumination and optical fluence attenuation in three-dimensional optoacoustic tomography of the breast,” Proc. SPIE 10878, 108784X (2019). PSISDG 0277-786X 10.1117/12.2514750

[39] Kim M. et al., “Correction of wavelength-dependent laser fluence in swept-beam spectroscopic photoacoustic imaging with a hand-held probe,” Photoacoustics 19, 100192 (2020). 10.1016/j.pacs.2020.100192 32670789 PMC7339128

[40] Sandell J. L. Zhu T. C., “A review of in-vivo optical properties of human tissues and its impact on PDT,” J. Biophotonics 4(11–12), 773–787 (2011). 10.1002/jbio.201100062 22167862 PMC3321368

[41] Richards-Kortum R. Sevick-Muraca E., “Quantitative optical spectroscopy for tissue diagnosis,” Annu. Rev. Phys. Chem. 47(1), 555–606 (1996). ARPLAP 0066-426X 10.1146/annurev.physchem.47.1.555 8930102

[42] Cheong W. F. Prahl S. A. Welch A. J., “A review of the optical properties of biological tissues,” IEEE J. Quantum Electron. 26(12), 2166–2185 (1990). IEJQA7 0018-9197 10.1109/3.64354

[43] Pickering J. W. et al., “Double-integrating-sphere system for measuring the optical properties of tissue,” Appl. Opt. 32(4), 399–410 (1993). APOPAI 0003-6935 10.1364/AO.32.000399 20802704

[44] Setchfield K. et al., “Relevance and utility of the in-vivo and ex-vivo optical properties of the skin reported in the literature: a review,” Biomed. Opt. Express 14(7), 3555–3583 (2023). BOEICL 2156-7085 10.1364/BOE.493588 37497524 PMC10368038

[45] Wilson B. C. Patterson M. S., “The physics of photodynamic therapy,” Phys. Med. Biol. 31(4), 327–360 (1986). PHMBA7 0031-9155 10.1088/0031-9155/31/4/001 3526361

[46] Wang L. V. Wu H.-i., Biomedical Optics: Principles and Imaging, John Wiley & Sons (2007).

[47] Wilson B. C. Adam G., “A Monte Carlo model for the absorption and flux distributions of light in tissue,” Med. Phys. 10(6), 824–830 (1983). MPHYA6 0094-2405 10.1118/1.595361 6656695

[48] Fang Q. Boas D. A., “Monte Carlo simulation of photon migration in 3D turbid media accelerated by graphics processing units,” Opt. Express 17(22), 20178–20190 (2009). OPEXFF 1094-4087 10.1364/OE.17.020178 19997242 PMC2863034

[49] Bashkatov A. N. et al., “Optical properties of human skin, subcutaneous and mucous tissues in the wavelength range from 400 to 2000 nm,” J. Phys. Appl. Phys. 38(15), 2543–2555 (2005). 10.1088/0022-3727/38/15/004

[50] Aernouts B. et al., “Visible and near-infrared bulk optical properties of raw milk,” J. Dairy Sci. 98(10), 6727–6738 (2015). JDSCAE 0022-0302 10.3168/jds.2015-9630 26210269

[51] Xia J. J. et al., “Characterizing beef muscles with optical scattering and absorption coefficients in VIS-NIR region,” Meat Sci. 75(1), 78–83 (2007). MESCDN 0309-1740 10.1016/j.meatsci.2006.07.002 22063414

[52] Jacques S. L., “Optical properties of biological tissues: a review,” Phys. Med. Biol. 58(11), R37–R61 (2013). PHMBA7 0031-9155 10.1088/0031-9155/58/11/R37 23666068

[53] Hosseindokht Z. et al., “Photoacoustic viscoelasticity assessment of prefrontal cortex and cerebellum in normal and prenatal valproic acid-exposed rats,” Photoacoustics 36, 100590 (2024). 10.1016/j.pacs.2024.100590 38318427 PMC10839762

[r54] Fung Y., “Elasticity of soft tissues in simple elongation,” Am. J. Physiol. 213(6), 1532–1544 (1967). AJPHAP 0002-9513 10.1152/ajplegacy.1967.213.6.1532 6075755

[r55] Skovoroda A. R. et al., “Theoretical analysis and verification of ultrasound displacement and strain imaging,” IEEE Trans. Ultrason. Ferroelectr. Freq. Control 41(3), 302–313 (1994). ITUCER 0885-3010 10.1109/58.285463

[r56] Cao R. et al., “Tissue mimicking materials for the detection of prostate cancer using shear wave elastography: a validation study,” Med. Phys. 40(2), 022903 (2013). MPHYA6 0094-2405 10.1118/1.4773315 23387774 PMC3562344

[r57] Lubinski M. A. Emelianov S. Y. O’Donnell M., “Speckle tracking methods for ultrasonic elasticity imaging using short-time correlation,” IEEE Trans. Ultrason. Ferroelectr. Freq. Control 46(1), 82–96 (1999). ITUCER 0885-3010 10.1109/58.741427 18238401

[r58] Tweten D. J. et al., “Estimation of material parameters from slow and fast shear waves in an incompressible, transversely isotropic material,” J. Biomech. 48(15), 4002–4009 (2015). JBMCB5 0021-9290 10.1016/j.jbiomech.2015.09.009 26476762 PMC4663187

[59] Rouze N. C. et al., “Finite element modeling of impulsive excitation and shear wave propagation in an incompressible, transversely isotropic medium,” J. Biomech. 46(16), 2761–2768 (2013). JBMCB5 0021-9290 10.1016/j.jbiomech.2013.09.008 24094454 PMC3879727

[60] Thomas A. et al., “Quantitative photoacoustic imaging using known chromophores as fluence marker,” Photoacoustics 41, 100673 (2025). 10.1016/j.pacs.2024.100673 39830068 PMC11741946

[61] Shao P. Cox B. Zemp R. J., “Estimating optical absorption, scattering, and Grueneisen distributions with multiple-illumination photoacoustic tomography,” Appl. Opt. 50(19), 3145–3154 (2011). APOPAI 0003-6935 10.1364/AO.50.003145 21743514

[62] Sowers T. Yoon H. Emelianov S., “Investigation of light delivery geometries for photoacoustic applications using Monte Carlo simulations with multiple wavelengths, tissue types, and species characteristics,” J. Biomed. Opt. 25(1), 016005 (2020). JBOPFO 1083-3668 10.1117/1.JBO.25.1.016005 31975577 PMC6976898

[63] Huda K. et al., “Towards transabdominal functional photoacoustic imaging of the placenta: improvement in imaging depth through optimization of light delivery,” Ann. Biomed. Eng. 49(8), 1861–1873 (2021). ABMECF 0090-6964 10.1007/s10439-021-02777-0 33909192 PMC8373763

[64] Zhang E. Laufer J. Beard P., “Backward-mode multiwavelength photoacoustic scanner using a planar Fabry–Perot polymer film ultrasound sensor for high-resolution three-dimensional imaging of biological tissues,” Appl. Opt. 47(4), 561 (2008). APOPAI 0003-6935 10.1364/AO.47.000561 18239717

[65] Cho S. et al., “An ultrasensitive and broadband transparent ultrasound transducer for ultrasound and photoacoustic imaging in-vivo,” Nat. Commun. 15(1), 1444 (2024). NCAOBW 2041-1723 10.1038/s41467-024-45273-4 38365897 PMC10873420

[66] Hochuli R. et al., “Estimating blood oxygenation from photoacoustic images: can a simple linear spectroscopic inversion ever work?” J. Biomed. Opt. 24(12), 121914 (2019). JBOPFO 1083-3668 10.1117/1.JBO.24.12.121914 31849203 PMC7005536

[67] Gröhl J. et al., “Learned spectral decoloring enables photoacoustic oximetry,” Sci. Rep. 11(1), 6565 (2021). SRCEC3 2045-2322 10.1038/s41598-021-83405-8 33753769 PMC7985523

[68] Falco I. Bossy E. Arnal B., “Imaging light fluence in blood vessels by combining photoacoustic fluctuation imaging and ultrasound power Doppler,” Phys. Med. Biol. 69(16), 165026 (2024). PHMBA7 0031-9155 10.1088/1361-6560/ad672e 39047777

[69] Rajian J. R. Carson P. L. Wang X., “Quantitative photoacoustic measurement of tissue optical absorption spectrum aided by an optical contrast agent,” Opt. Express 17(6), 4879–4889 (2009). OPEXFF 1094-4087 10.1364/OE.17.004879 19293919 PMC2689517

[70] Huang X. et al., “Photoacoustic-strain (PAS) imaging for tissue microcirculation assessment,” IEEE Trans. Med. Imaging (2025). 10.1109/TMI.2025.3562141 PMC12392290 40249682

