# Supplementary material for: Depth-dependent fluence compensation without a priori knowledge of tissue composition for quantitative ultrasound-guided photoacoustic imaging
Source: J Biomed Opt. 2025 Jul 12;30(7):076005. doi: 10.1117/1.JBO.30.7.076005 (PMC12255355; doi:10.1117/1.JBO.30.7.076005)
Supplement: Supplementary file 1 [file JBO_030_076005_SD001.pdf]

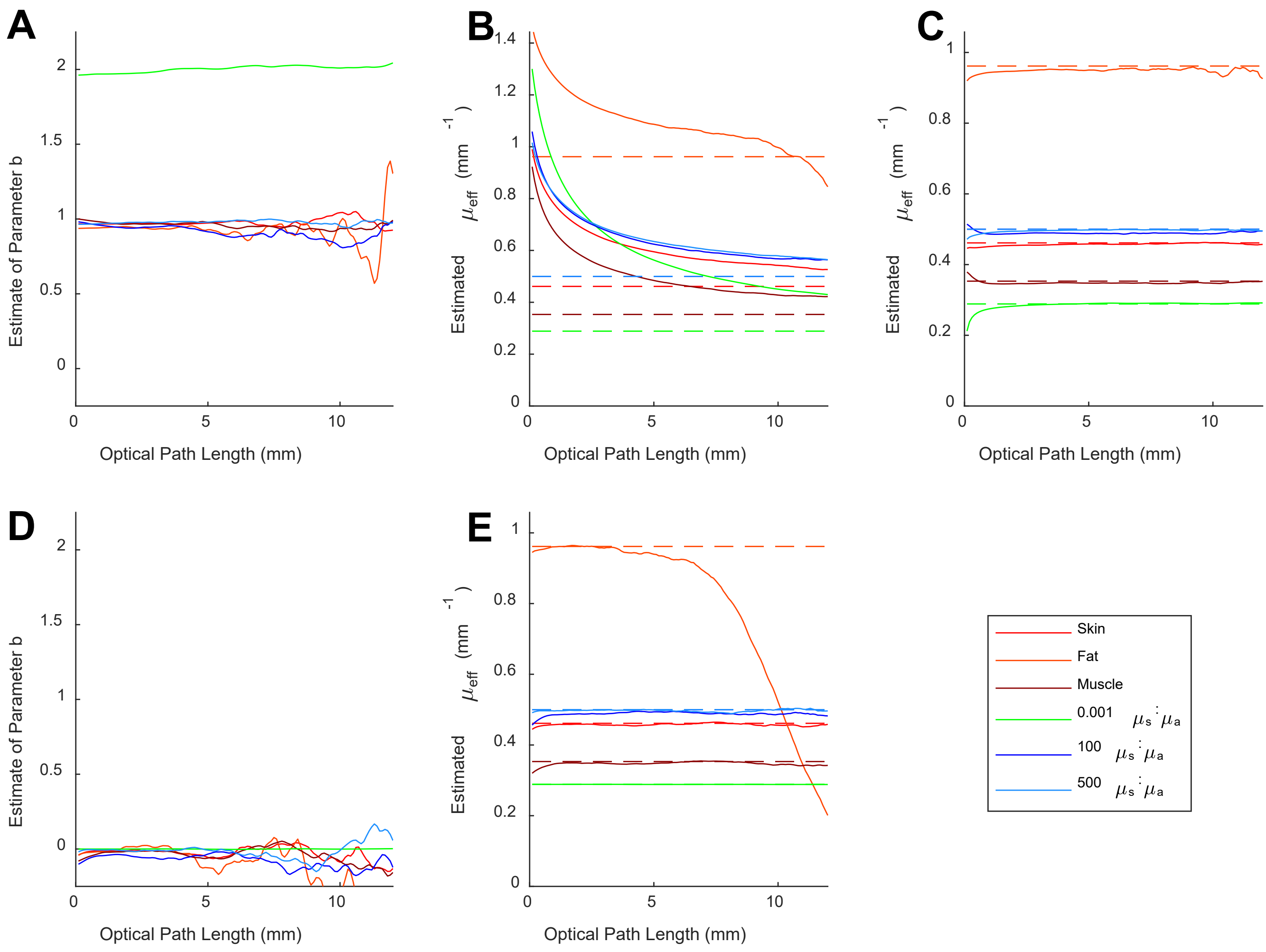

Fig. S1 Monte Carlo simulation results of ideal isotropic point source and semi-infinite plane wave: (A) Estimated value of geometry parameter as a function of distance from isotropic light source, showing that biological tissues and high μ_s: μ_a ratios converge as expected to $b = 1$, while the absorption-dominant case converges to $b = 2$. (B) Estimation of μ_{eff} without compensation for geometry shows over-estimate (solid lines) compared to the ground-truth (dotted lines). (C) Estimation of μ_{eff} with geometry compensation applied, showing correct estimation for all cases. (D) Estimated value of geometry parameter as a function of distance from semi-infinite plane source, showing that all cases converge as expected to $b = 0$. (E) Estimation of μ_{eff} for a plane source requires no compensation for geometry as plane waves do not undergo geometric spreading.

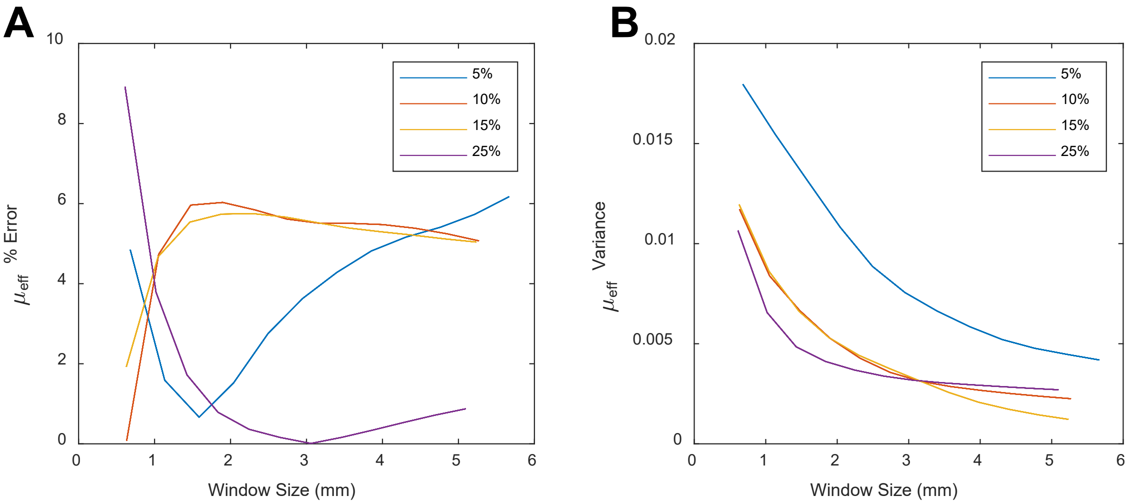

Fig. S2 (A) Effect on μ_{eff} estimation error for phantom experiment in milk/water mixture is found for different sizes of moving window over which μ_{eff} is calculated. (B) Effect on μ_{eff} variance for milk phantom experiment is found for different sizes of moving window over which μ_{eff} is calculated.

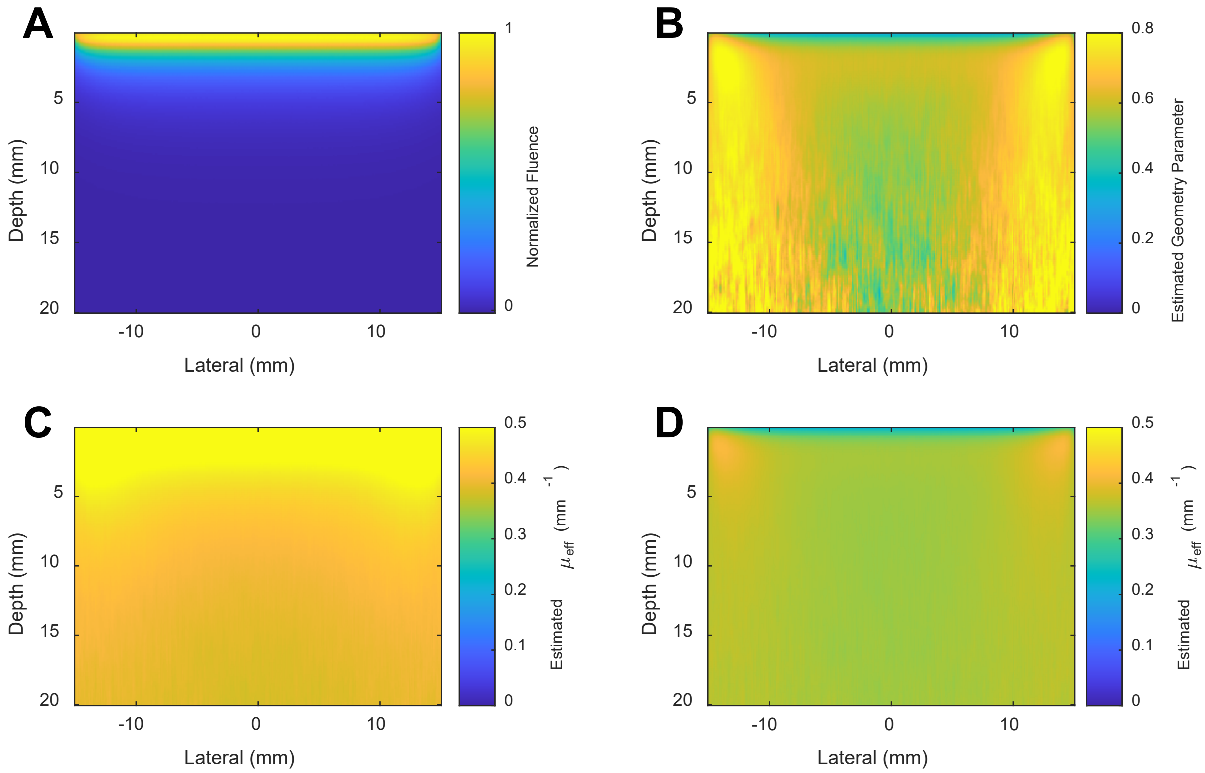

Fig. S3 Monte Carlo simulation results of rectangular 30 mm x 2 mm aperture in homogeneous volume of muscle tissue at 700 nm, showing the imaging plane: (A) Fluence pattern emanating from the rectangular aperture. (B) Geometry parameter varies laterally, but is consistent within the middle 15 mm of the field-of-view. (C) Without correction for geometry, estimated μ_{eff} (mm^{-1}) is higher than the ground truth (0.35 mm^{-1}). (D) Correcting by the geometry parameter in the center of the field-of-view restores accurate estimation of μ_{eff} (mm^{-1}) in the center.

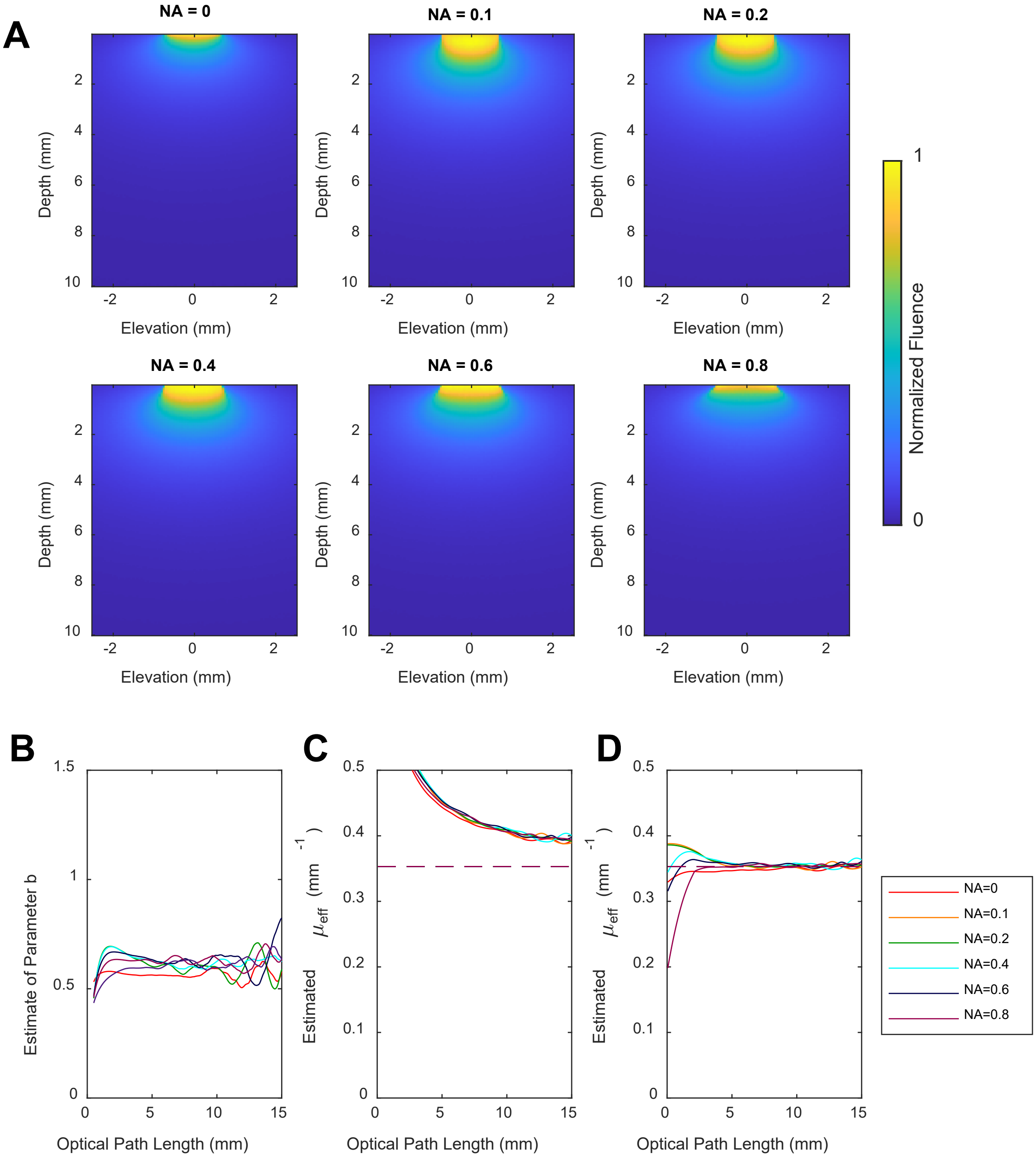

Fig. S4 Monte Carlo simulation results of rectangular 30 mm x 2 mm aperture with varying numerical aperture (NA) in homogeneous volume of muscle tissue at 700 nm, showing the axial-elevational plane: (A) Fluence pattern emanating from the rectangular aperture in the elevational direction changes with increasing NA. (B) Geometry parameter undergoes slight increase with increasing NA. (C) Without correction for geometry, estimated μ_{eff} is higher than the ground truth (0.35 mm^{-1}). (D) Correcting by the geometry parameter restores accurate estimation of μ_{eff} for all NA.

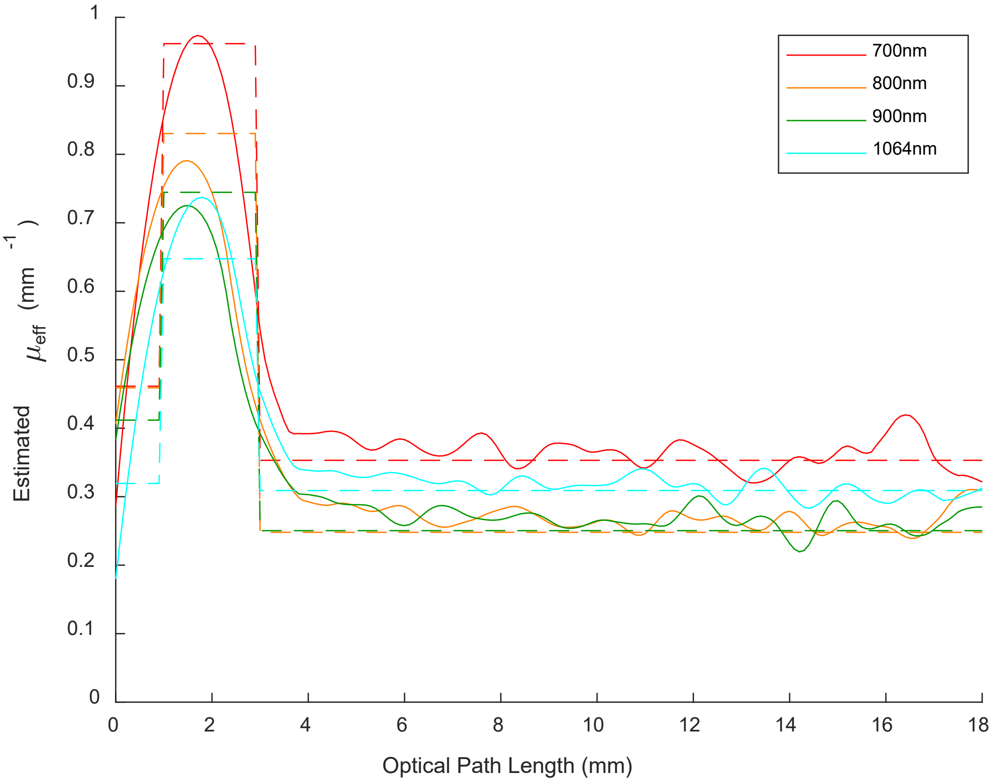

Fig. S5 Estimation of μ_{eff} in a Monte Carlo simulation of a multi-layered tissue volume of skin, fat, and muscle tissue at four different wavelengths, showing estimates (solid lines) that follow the ground truth values of respective tissue layers (dotted lines). Additionally, μ_{eff} can be accurately estimated for four different wavelengths relevant to PA imaging.
